# Monocyte deactivation in neutropenic acute respiratory distress syndrome patients treated with granulocyte colony-stimulating factor

**DOI:** 10.1186/cc6791

**Published:** 2008-02-18

**Authors:** Djamel Mokart, Eric Kipnis, Pierre Guerre-Berthelot, Norbert Vey, Christian Capo, Antoine Sannini, Jean-Paul Brun, Jean-Louis Blache, Jean-Louis Mege, Didier Blaise, Benoit P Guery

**Affiliations:** 1 Department of Anesthesiology and Intensive Care Unit, Paoli-Calmette Institute, 232 bd Sainte Marguerite, 13273 Marseille Cedex 9, France; 2 EA2689, SGRIVI, Hopital Calmette, CHRU de Lille, 59037 Lille Cedex; 3 Laboratory of Immunology and Hematology, Hôpital de la Conception, Marseille, France; 4 Department of Hematology, Paoli-Calmette Institute, Marseille, France

## Abstract

**Introduction:**

In severely neutropenic septic acute respiratory distress syndrome (ARDS) patients, macrophages and monocytes are the last potentially remaining innate immune cells. We have previously shown, however, a deactivation of the alveolar macrophage in neutropenic septic ARDS patients. In the present study, we tried to characterize *in vitro *monocyte baseline cytokine production and responsiveness to lipopolysaccharide exposure.

**Methods:**

Twenty-two consecutive patients with cancer were prospectively enrolled into a prospective observational study in an intensive care unit. All patients developed septic ARDS and were divided into two groups: neutropenic patients (*n *= 12) and non-neutropenic patients (*n *= 10). All of the neutropenic patients received granulocyte colony-stimulating factor whereas no patient in the non-neutropenic group received granulocyte colony-stimulating factor. We compared monocytes from neutropenic patients with septic ARDS with monocytes from non-neutropenic patients and healthy control individuals (*n *= 10). Peripheral blood monocytes were cultured, and cytokine levels (TNFα, IL-1β, IL-6, IL-10, and IL-1 receptor antagonist) were assayed with and without lipopolysaccharide stimulation.

**Results:**

TNFα, IL-6, IL-10 and IL-1 receptor antagonist levels in unstimulated monocytes were lower in neutropenic patients compared with non-neutropenic patients. Values obtained in the healthy individuals were low as expected, comparable with neutropenic patients. In lipopolysaccharide-stimulated monocytes, both inflammatory and anti-inflammatory cytokine production were significantly lower in neutropenic patients compared with non-neutropenic patients and control individuals.

**Conclusion:**

Consistent with previous results concerning alveolar macrophage deactivation, we observed a systemic deactivation of monocytes in septic neutropenic ARDS. This deactivation participates in the overall immunodeficiency and could be linked to sepsis, chemotherapy and/or the use of granulocyte colony-stimulating factor.

## Introduction

The role of the host immune response in the pathogenesis of septic acute respiratory distress syndrome (ARDS) remains unclear. Indeed, cytokine-producing activated inflammatory cells recruited to the lung are the major determinant of the innate immune defense to respiratory pathogens [1]. The impairment of the response facilitates infection and therefore pathogen-mediated injury [1].

In patients severely neutropenic from exposure to radiation or cytotoxic drugs, the recruitment of neutrophils into the lung is an evidently impaired defense mechanism. In these patients, several other cellular populations taking part in the innate immune response may remain available.

One alternative population may be activated alveolar macrophages, which can release a wide variety of mediators [2-5]. We recently demonstrated, however, a deactivation of alveolar macrophages in neutropenic patients with ARDS [6]. Another alternative population could be monocytes, whose role and state of activation remains unclear in septic ARDS – although several studies have found evidence of monocyte deactivation in human sepsis [7,8].

Our hypothesis, therefore, was that monocytes could play a major role, in addition to neutropenia, in the immunosuppression of neutropenic patients treated with granulocyte colony-stimulating factor (G-CSF) and presenting septic ARDS. The objective of our study was to find evidence of monocyte hyporeactivity in these patients.

To characterize monocyte hyporeactivity, we evaluated monocyte cytokine production *in vitro *under basal conditions and after lipopolysaccharide (LPS) exposure, using cultured monocytes isolated from the blood of neutropenic patients treated with G-CSF or non-neutropenic patients, both presenting septic ARDS. We also used healthy individuals' monocytes as a control population.

## Patients and methods

### Patients

Twenty-two consecutive patients with cancer were prospectively enrolled in the study. All patients had developed documented septic ARDS and were divided into two groups: neutropenic patients (absolute neutrophil count <1,000/mm^3^) treated with G-CSF, and non-neutropenic patients (absolute neutrophil count >1,000/mm^3^).

We used the definition of ARDS recommended by the American–European Consensus Conference [9]. Sepsis was defined according to the criteria of the American College of Chest Physicians/Society of Critical Care Medicine Consensus Conference [10]. The study was conducted after obtaining approval from our institutional Ethics Committee; informed consent was obtained from each patient's next of kin or directly from the healthy volunteers.

Standard supportive cares as well as broad-spectrum antibiotics were provided for each patient. All neutropenic patients were treated with G-CSF prior to intensive care unit admission, whereas no patient received G-CSF in the non-neutropenic patient group. All patients underwent blood sampling during the first 3 days after the onset of ARDS. The duration of ARDS prior to monocyte harvesting was similar in neutropenic patients and non-neutropenic patients.

### Isolation and culture of monocytes

Ten milliliters of blood were sampled, diluted in isotonic saline and were centrifuged. The cellular pellet containing mononuclear cells was recovered, and monocytes were isolated by plastic adherence and incubated with supplemented RPMI 1640 (10% fetal calf serum, 2 mM L-glutamate, 100 U/ml penicillin, 100 mg/ml streptomycin) for 24 hours at 37°C. Endotoxin contamination was excluded by testing reagents with the Limulus amebocyte lysate assay (Whittaker Bioproducts, Fontenay-sous-Bois, France).

### Monocyte activation and immunoassays for cytokine determination

Monocytes were cultured (5 × 10^5 ^cells/assay) in RPMI 1640 containing 10% fetal bovine serum and antibiotics in the presence or the absence of LPS (*Escherichia coli *extract, 055:B5; Sigma, St Louis, MO, USA) for 16 hours at 37°C. Cell supernatants were collected and stored at -70°C before assays. Cytokine measurements were performed with a quantitative sandwich enzyme immunoassay (R&D Systems, British Biotechnology, Abingdon, UK). Supernatants were assayed for TNFα, IL-1β, IL-6 and IL-10 with enzyme immunoassays provided by Immunotech (Marseille, France). IL-1 receptor antagonist was assayed using a kit provided by R&D Systems. The intra-assay and inter-assay coefficients of variation of the immunoassay kits ranged between 5% and 10%.

### Statistical analysis

Comparisons between two independent groups were analyzed by the Mann–Whitney U test. For dichotomous data, percentages were calculated and were compared using Fisher's exact test. To assess whether cytokine production was modified in each subgroup under the two conditions (baseline and LPS stimulation), a Wilcoxon paired data statistical test was performed. For each test, *P *< 0.05 was considered significant.

## Results

The baseline characteristics of the neutropenic patients and non-neutropenic patients are summarized in Table 1.

**Table 1:** Medical characteristics of the patients

Patient	Hematologic disease	Cause of ARDS	Cancer status	Stem cell transplantation	Chemotherapy	Neutropenia and G-CSF treatment
1	Acute myeloid leukemia	Bacterial pneumonia	Newly diagnosed	No	Cytosine arabinoside + daunorubicin	Yes
2	Hodgkin's disease	Bacterial pneumonia	Complete remission	Peripheral autologous blood stem cell transplantation	Vincristin + bleomycin + doxorubicin	Yes
3	Non-Hodgkin's lymphoma	Bacteremia	Newly diagnosed	No	Cyclophosphamide + methotrexate + vincristin + doxorubicin	Yes
4	Acute myeloid leukemia	Fungal pneumonia	Newly diagnosed	No	Cytosine arabinoside + daunorubicin	Yes
5	Non-Hodgkin's lymphoma	Bacterial pneumonia	Complete remission	Allogeneic hematopoietic stem cells	Cyclophosphamide + vincristin + doxorubicin	Yes
6	Acute myeloid leukemia	Bacterial pneumonia	Relapse	Allogeneic hematopoietic stem cells	Cyclophosphamide + methotrexate + cytosine arabinoside + vincristin + daunorubicin	Yes
7	Non-Hodgkin's lymphoma	Septic shock	Complete remission	Peripheral autologous blood stem cell transplantation	Cyclophosphamide + doxorubicin	Yes
8	Chronic lymphocytic leukemia	Septic shock	Partial remission	Peripheral autologous blood stem cell transplantation	Cyclophosphamide + doxorubicin	Yes
9	Acute lymphoblastic leukemia	Fungal pneumonia	Newly diagnosed	No	Cyclophosphamide + daunorubicin + vincristin	Yes
10	Non-Hodgkin's lymphoma	Bacterial pneumonia	Newly diagnosed	No	Cyclophosphamide + methotrexate + vincristin + doxorubicin	Yes
11	Hodgkin's disease	Septic shock	Complete remission	No	Vincristin + bleomycin + doxorubicin	No
12	Thymoma	Bacterial pneumonia	Complete remission	Peripheral autologous blood stem cell transplantation	Melphalan	No
13	Esophageal cancer	Bacterial pneumonia	Complete resection	No	Surgery	No
14	Acute myeloid leukemia	Fungal pneumonia	Newly diagnosed	No	Cytosine arabinoside + daunorubicin	No
15	Rectal cancer	Viral pneumonia	Complete resection	No	Surgery	No
16	Breast cancer	Candidemia	Complete resection	No	Surgery + epirubicin + taxotere	No
17	Esophageal cancer	Bacterial pneumonia	Complete resection	No	Surgery	No
18	Stomach cancer	Bacterial pneumonia	Complete resection	No	Surgery	No
29	Esophageal cancer	Mediatinis	Complete resection	No	Surgery	No
20	Esophageal cancer	Bacterial pneumonia	Complete resection	No	Surgery	No
21	Breast cancer	Bacterial pneumonia	Complete resection	No	Surgery + epirubicin + taxotere	No
22	Bladder cancer	Bacterial pneumonia	Complete resection	No	Surgery	No

ARDS = acute respiratory distress syndrome; G-CSF = granulocyte colony-stimulating factor.

Table 2 presents the monocyte cytokine production under basal conditions and after LPS exposure, in cultured monocytes isolated from the blood of neutropenic patients with ARDS or from non-neutropenic septic ARDS patients and healthy control individuals.

**Table 2:** Cytokine production at baseline and after lipopolysaccharide induction for neutropenic patients and for non-neutropenic patients and control individuals

Cytokine	Control individuals (*n *= 10)	Non-neutropenic patients (*n *= 12)	Neutropenic patients (*n *= 10)
Spontaneous production			
TNFα	25 (10–50)	100 (10–400)^*†^	10 (10–10)
IL-1	20 (15–45)	30 (15–870)	15 (15–15)
IL-6	85 (10–115)	5,580 (20–19,560)^*†^	45 (9–61)
IL-1 receptor antagonist	205 (90–920)	5,740 (430–9,660)^*†^	175 (74–713)
IL-10	15 (5–45)	50 (5–870)^*†^	5 (5–5)
Lipopolysaccharide-induced production			
TNFα	280 (125–970)^*‡^	1,830 (560–3,570)^*†‡^	10 (10–10)
IL-1	855 (105–1,550)^*‡^	7,900 (410–9,120)^*†‡^	15 (15–15)
IL-6	6205 (520–8,825)^*‡^	53,720 (13,800–79,450)^*†‡^	720 (3–1,113)
IL-1 receptor antagonist	1045 (340–2,250)^*‡^	19,110 (5,785–39,110)^*†‡^	335 (180–780)
IL-10	305 (70–805)^*‡^	1,875 (290–3,280)^†‡^	5 (5–5)

Data (pg/ml) are expressed as the median and quartile (25th–75th percentiles). *P *< 0.05 was considered significant: *P < 0.05 versus neutropenic patients, using the Mann–Whitney U test; ^†^*P *< 0.05 versus control individuals, using the Mann–Whitney U test; and ^‡^*P *< 0.05 baseline versus lipopolysaccharide conditions, using the Wilcoxon test.

In monocytes from healthy control individuals, the cytokine levels – both proinflammatory and anti-inflammatory – increased significantly from baseline levels in response to LPS, demonstrating the reactivity of normal monocytes. Monocytes from non-neutropenic septic ARDS patients showed a similar response, albeit from significantly higher baseline levels than control individuals (except for IL-1). There was a significant increase in all cytokines in response to LPS – reaching levels that were significantly higher than those of control individuals, demonstrating an increased reactivity of these monocytes. Monocytes from neutropenic septic ARDS patients, however, showed baseline levels similar to control individuals, demonstrating a suppressed state. These levels did not increase in response to LPS and were therefore significantly lower than LPS-responsive monocytes from control individuals and non-neutropenic septic ARDS patients, demonstrating the hyporeactivity of these monocytes.

The analysis of available clinical data showed that the mortality rate was significantly higher in neutropenic patients than in non-neutropenic patients, although the PaO_2_/FiO_2 _ratio and the Simplified Acute Physiology Score II were comparable between the two groups (Table 3).

**Table 3:** Demographic data

Parameter	Neutropenic patients	Non-neutropenic patients
*n*	10	12
Simplified Acute Physiology Score II	45 (36–57)	38 (34–62)
Age (years)	50 (41–64)	48 (38–64)
PaO_2_/FiO_2 _(mm/Hg)	160 (150–180)	150 (129–176)
Sex (male/female)	7/3	9/3
Mortality rate (%)	80	42*
Duration of neutropenia before ARDS (days)	3 (1–7)	
Duration of neutropenia during ARDS (days)	5 (2–10)	
Duration of treatment by G-CSF before ARDS (days)	5 (3–9)	
Duration of sepsis before monocyte harvesting (days)	3 (0–5)	3 (1–6)
Duration of sepsis before ARDS diagnosis (days)	2 (0–5)	3 (1–5)

Data are expressed as the median and quartile (25th–75th percentiles), numbers or percentages. ARDS = acute respiratory distress syndrome; G-CSF = granulocyte colony-stimulating factor. *P < 0.05 for neutropenic patients versus non-neutropenic patients using Fisher's exact test.

## Discussion

Our results show that cultured monocytes isolated from the blood of neutropenic septic ARDS patients are suppressed, as evidenced by low baseline cytokine levels, and are hyporeactive in response to LPS stimulation, as evidenced by an absence of cytokine increase, compared with monocytes from non-neutropenic septic ARDS patients and healthy control individuals.

These results correlate with our previous study suggesting that neutropenic patients with septic ARDS exhibited a local pulmonary immunosuppression related to alveolar macrophage deactivation [6], but also exhibited a deeply hyporeactive monocytic/macrophagic response. Indeed, we had previously shown in these patients that bronchoalveolar lavage had a low cellularity with a predominance of alveolar macrophages. HLA-DR was downregulated and the LPS-induced macrophagic cytokine response was decreased, both of these features underlining a severe hyporeactive state. This local macrophage hyporeactivity was associated with a state of severe alveolar neutropenia contributing to local host immunosuppression. The systemic monocytic hyporeactivity found in the present study can only contribute to this host immunosuppression.

Since all neutropenic patients exhibited a profound leukopenia (leukocyte count <100/mm^3^) we were not able to evaluate monocyte HLA-DR expression using a flow cytometer (data not shown). Speculating that our results are in line with previous evidence of a systemic deactivation of monocyte antigen or leukocytes described in septic patients [11], however, is tempting. Monocyte deactivation evidenced by loss of HLA-DR has been shown to occur early in septic patients and persisting deactivation correlates with mortality [7]. Leukocyte hyporesponsiveness in cytokine production to LPS has been shown in sepsis due to both Gram-negative and Gram-positive pathogens [8]. A more complete phenotypic and functional characterization of monocyte deactivation, termed immunoparalysis, has been described in which loss of HLA-DR and cytokine hyporesponsiveness are still the main components [11].

The mechanisms of such a hyporeactive state are unclear and are probably multifactorial, related to sepsis [12], chemotherapy [13] or neutropenia *per se*. First of all, the evolution of sepsis follows a biphasic immunological pattern: the early hyperinflammatory phase is counterbalanced by an anti-inflammatory response that may lead to a hypoinflammatory, immunosuppressed state [12]. This latter condition is associated with immunodeficiency and monocyte deactivation. Associated with sepsis, chemotherapy can induce a downregulation of cytokine production through a direct cytotoxic effect [13]. Interestingly, the decrease of cytokine production can occur regardless of the drugs used, suggesting a nonspecific mechanism [13].

We cannot, however, consider these effects of a decrease in cytokines due to monocyte hyporeactivity on the host immune status in septic ARDS patients without discussing their potential involvement in ARDS. Indeed, cytokine production by innate immune response cells is a double-edged sword: impairment leads to immune suppression and infection-related injury but an increased or decompartmentalized proinflammatory cytokine response is a major component of ARDS [1], and several studies have shown that, in ARDS or pneumonia, an increased inflammatory response could be associated with a worse outcome [14,15]. Furthermore, anti-inflammatory cytokines are involved in lung repair modulation following injury [4].

In the present study, the monocyte deactivation observed in neutropenic patients compared with non-neutropenic patients concerned both proinflammatory and anti-inflammatory cytokines. Given the nonexhaustive panel of measured cytokines and the impossibility of relating measured cytokine levels to a net proinflammatory or anti-inflammatory balance, it is impossible to conclude whether the observed hyporeactivity leads to a proinflammatory or anti-inflammatory imbalance.

Although our study was not designed to study the relationship between monocyte deactivation and mortality, we observed an increased mortality in neutropenic ARDS patients (hyporeactive monocytes) compared with non-neutropenic patients with ARDS (hyperreactive), unexplained by ARDS severity or patient severity since there was no difference in the PaO_2_/FiO_2 _ratio or the Simplified Acute Physiology Score II between these groups.

While local immunosuppression is associated with a good prognosis in non-neutropenic patients with ARDS [16], a systemic immunosuppression (monocyte deactivation, neutropenia) could be inappropriate in neutropenic patients with septic ARDS and could participate in increased mortality.

All neutropenic patients in the present study received G-CSF as a supportive treatment – a molecule that induces anti-inflammatory cytokine secretion [17] and may increase monocyte deactivation – so the observed low-LPS-stimulated production in neutropenic patients could be related to the use of G-CSF. This balance between proinflammatory and anti-inflammatory response is extremely variable depending on the trigger. In the study of Hartung and colleagues, the authors demonstrate that the major effect of G-CSF was a shift toward an anti-inflammatory cytokine response; in fact, a reduction in TNF release was obtained with various stimuli except LPS [17]. The consequences of G-CSF may also influence the evolution of the acute lung injury. In a rat model of acute lung injury during neutropenia, Azoulay and colleagues showed an increase of alveolar cell recruitment and pulmonary edema in G-CSF-treated animals [18]. The authors concluded in their study that neutropenia recovery could worsen acute lung injury, with an exacerbation by G-CSF. Moreover, in a clinical study, Karlin and colleagues also showed that G-CSF-induced neutropenia recovery was associated with a risk of respiratory status deterioration [19]. Furthermore, G-CSF could promote the development of ARDS due to pulmonary infections in neutropenic patients [20]. From these data, the use of G-CSF in this situation remains to be carefully evaluated. Since all patients received G-CSF in our study, however, there was no bias because of its use.

Our study has several limitations. We chose to use a control population with solid organ malignancies; among these few had undergone chemotherapy, but on the contrary the neutropenic patients all received chemotherapy. It is therefore possible that chemotherapeutic agents may account for some differences in cytokine production. In the present study we did not analyze other parameters reflecting monocyte functions, such as phagocytic engulfment, peptide recognition or migration; as a consequence, we cannot rule out other aspects of monocyte function being preserved. Finally, we did not study cell surface epitopes that may also be involved in the decreased production of cytokines in neutropenic patients.

If there is, as we can assume from our results, a parallel between alveolar and systemic cellular response, a monitoring of lung defense mechanisms could be proposed through the study of baseline and LPS-induced cytokine production over time in monocytes isolated from blood samples. Additionally, measurement of circulating concentrations of inflammatory mediators could be useful to evaluate, and possibly target, the best time for the administration of immunomodulating agents [21].

The present findings contribute to the debate on the use of anti-inflammatory treatment in the management of sepsis. A better understanding of what is the adequate balance of anti-inflammatory cytokines to proinflammatory cytokines is probably the key to the improvement of neutropenic septic patients.

## Key messages

• Neutropenic patients treated with G-CSF and presenting septic ARDS show monocyte deactivation.

• This hyporeactive state could be related to sepsis, chemotherapy or neutropenia.

• The use of G-CSF in this situation remains to be carefully evaluated.

## Abbreviations

ARDS = acute respiratory distress syndrome; G-CSF = granulocyte colony-stimulating factor; HLA-DR = class II major histocompatibility complex; IL = interleukin; LPS = lipopolysaccharide; PaO_2_/FiO_2 _= PaO_2_/fraction of inspired oxygen; TNF = tumor necrosis factor.

## Competing interests

The authors declare that they have no competing interests.

## Authors' contributions

DM, AS, CC, BPG and EK collected and analyzed the data. DM, J-PB, J-LB, DB, J-LM and PGB reviewed and coordinated the study.
